# Simultaneous Oculomotor and Facial Nerve Palsies in a Patient with Systemic Lupus Erythematosus and Sjögren's Syndrome

**DOI:** 10.1155/2019/4156781

**Published:** 2019-04-11

**Authors:** Natsuki Shima, Takamasa Murosaki, Shotaro Yamamoto, Akiko Miyanaga, Hiroi Kusaka, Katsuya Nagatani, Takao Nagashima, Mikio Sawada, Masahiro Iwamoto, Seiji Minota

**Affiliations:** ^1^Division of Rheumatology and Clinical Immunology, Department of Medicine, Jichi Medical University, Shimotsuke, Tochigi, Japan; ^2^Department of Neurology, Haga Red Cross Hospital, Mooka, Tochigi, Japan

## Abstract

A 70-year-old man with systemic lupus erythematosus (SLE) presented with simultaneous right oculomotor nerve palsy and right facial nerve palsy. Brain magnetic resonance imaging and cerebrospinal fluid analysis revealed no abnormality. Coexistent Sjögren's syndrome was diagnosed on the basis of anti-SS-A antibody positivity, salivary gland scintigraphy, and histological findings on minor salivary gland biopsy. As there was no obvious cause of multiple cranial neuropathies, we supposed that the palsies were induced by either of the underlying diseases. The patient was treated with a high-dose of prednisolone and intravenous cyclophosphamide, and both palsies recovered almost completely within two weeks.

## 1. Introduction

Neuropsychiatric systemic lupus erythematosus (NPSLE) is one of the most complex expressions of systemic lupus erythematosus (SLE), which can affect the central, peripheral, and autonomous nervous systems [1]. Although the underlying mechanisms are largely not yet understood, several pathophysiological mechanisms have been identified, such as antibody-mediated neurotoxicity, vasculopathy due to anti-phospholipid antibodies, and cytokine-induced neurotoxicity [2].

Cranial nerve disturbance is one of the rare manifestations in NPSLE. The most frequent cranial neuropathies involve the optic nerve, the eighth nerve, the nerves responsible for the extraocular motor functions of the eye (third, fourth, and sixth), and less commonly, the ﬁfth and seventh nerves [3]. A recent review of NPSLE indicated that cranial neuropathy is a rare manifestation (0.5–1%) [4]. Several dozen case reports on peripheral cranial nerve palsy have been reported in patients with SLE [5–7].

Simultaneous occurrence of multiple cranial nerve palsies is rare in patients with SLE, except for brainstem involvement [8–10]. Here, we describe a patient with SLE who developed peripheral right oculomotor palsy and right facial nerve palsy simultaneously. After investigation, the patient was also found to have Sjögren's syndrome (SS).

## 2. Case Report

A 70-year-old man was admitted to our hospital with a two-week history of diplopia and right ptosis. He had a history of hypertension and dyslipidemia. Five months before admission, he had been diagnosed with SLE, according to the 1997 American College of Rheumatology classification criteria [11]. Because his arthritis and bicytopenia were mild, treatment with glucocorticoid had been withheld.

Physical examinations revealed ptosis of the right eye. The position of the right eye was laterally deviated. The adduction and upward and downward movements were reduced. Other significant features were mild drooping of the right angle of the mouth, incomplete closure of his right eye, and asymmetrical crease of the forehead. His hearing was normal, and there was no rash on the ear. Consciousness was clear, and the muscle strength of the extremities was normal.

Laboratory findings were as follows: white blood cell count, 2,200/*μ*L (neutrophils 900/*μ*L and lymphocytes 600/*μ*L); red blood cell count, 373 × 10^4^/*μ*L; hemoglobin, 10.9 g/dL; platelet count, 9.5 × 10^4^/*μ*L; and activated partial thromboplastin time, 32.4 sec. The blood glucose level was 99 mg/dL and HbA1c was 6.4%. C-reactive protein was 0.69 mg/dL, serum IgG was 4,450 mg/dL, and complement components C3 and C4 were 25 and 1 mg/dL, respectively. Serologic markers for hepatitis B and C were negative. Cryoglobulin was not detected. Rheumatoid factor was 18.1 IU/mL (normal <15), and antinuclear antibody was 1 : 2,560 with a homogeneous pattern. Anti-dsDNA antibody was 311.7 U/mL (normal <12), and anti-SS-A antibody was positive at a titer of 1 : 4. Anti-cardiolipin antibody (IgG) was positive at 23.8 U/mL (normal <10). Anti-U1-RNP, anti-Sm, anti-SS-B, anti-cardiolipin (IgM), and anti-*β*_2_ glycoprotein I antibodies and lupus anticoagulant were all negative. Urinalysis was normal. Cerebrospinal fluid showed no elevation of cell count or protein. Gadolinium-enhanced magnetic resonance imaging of the brain revealed no increased signal intensity on diffusion-weighted images, and there was no abnormal enhancement of cranial nerves or soft tissue mass around the nerves.

The patient had also noticed a dry mouth. A chewing gum test and salivary gland scintigraphy revealed decreased salivary secretion. Schirmer's test was only positive in his left eye. A labial minor salivary gland biopsy demonstrated focal lymphocytic infiltrations, which was consistent with the finding of SS. The patient was also diagnosed with SS according to the Japanese diagnostic criteria [12].

Because the patient was elderly, and had a history of hypertension and dyslipidemia, we first considered several common causes of cranial neuropathies including stroke, diabetes, and infections like varicella-zoster virus. However, these conditions did not explain the cranial nerve palsies. Hence, we considered that the cranial nerve palsies were caused by autoimmune mechanisms. The patient was treated with prednisolone 70 mg/day (1 mg/kg), aspirin, and intravenous cyclophosphamide pulse therapy. Thereafter, both the oculomotor and the facial nerve palsies began to improve in a few days. Both palsies recovered almost completely after two weeks of treatment.

## 3. Discussion

The present patient with SLE developed right oculomotor nerve palsy and right facial nerve palsy simultaneously. Although subjective symptoms were mild, SS was also diagnosed. Because we could not find any causes that could explain the simultaneous development of two different cranial nerve palsies, the cranial nerve palsies due to underlying diseases (SLE or SS) were diagnosed by exclusion. The treatment response was good with a high dose of prednisolone and intravenous cyclophosphamide.

Cranial nerve palsy occurs in patients with SS as well as SLE. It is also rare, except for trigeminal nerve involvement [13–16]. The frequency of cranial neuropathy ranges between 5% and 50% among patients with primary SS who have neurological symptoms [13–18]. The frequency of cranial neuropathy may be higher in Japanese SS patients (22–50%) than European SS patients (5–20%). Neurological manifestations of SS often precede the diagnosis of SS, and there are no sicca symptoms in one-third to half of patients with SS-associated neuropathy [14, 15]. Cranial neuropathy has also been reported as an initial manifestation of SS, even without sicca symptoms [19, 20].

In contrast to SLE, cases of multiple cranial nerve palsies have been reported in patients with SS [14, 21–23]. SS with facial nerve palsy often involves other cranial nerve involvement [14, 21]. It is difficult to discern which of the two underlying diseases was responsible for the multiple cranial nerve palsies in the present patient. However, we consider that the neuropathies are more likely to be due to SS than SLE for the following reasons. First, multiple cranial nerve palsies are more common in SS than SLE. Second, neurological manifestations of SS often precede the diagnosis of SS, as with the present patient. Third, the clinical symptoms of SLE were almost absent in this patient at the onset of neuropathies.

The diagnosis of multiple cranial neuropathies due to underlying diseases was made by excluding other possible causes. There are many causes of multiple cranial neuropathies due to brainstem lesion or extramedullary etiologies [24]. However, many causes can be excluded by adequate radiologic and cerebrospinal fluid studies. Oculomotor and facial nerve palsies can develop in diabetes mellitus [25]. The present patient was also suspected of having undiagnosed diabetes as one of the causes of cranial neuropathies. However, retro-orbital pain accompanies about half of the patients with diabetic oculomotor nerve neuropathy, and it is not particularly responsive to glucocorticoid therapy [26]. The present patient did not have orbital pain, and the response to glucocorticoid was swift. Generally, cranial neuropathy associated with SS shows good response to glucocorticoid therapy [14, 19, 20, 23, 27].

In conclusion, we encountered the case of an elderly patient with SLE and SS who developed oculomotor and facial nerve palsies simultaneously. In patients who have multiple cranial nerve palsies, brainstem lesions and extramedullary causes should be carefully ruled out. If these causes are ruled out, underlying subclinical SS should be explored because neurological symptoms can be the initial manifestation of SS, even without sicca symptoms.
